# Chemotherapeutic Effectiveness of Combining Cetuximab for Metastatic Colorectal Cancer Treatment: A System Review and Meta-Analysis

**DOI:** 10.3389/fonc.2020.00868

**Published:** 2020-05-28

**Authors:** Rong Li, Minqing Liang, Xiao Liang, Lu Yang, Min Su, Keng Po Lai

**Affiliations:** ^1^Guangxi Key Laboratory of Tumor Immunology and Microenvironmental Regulation, Guilin Medical University, Guilin, China; ^2^Department of Pharmacy, Guigang City People's Hospital, The Eighth Affiliated Hospital of Guangxi Medical University, Guigang, China; ^3^Department of Chemistry, City University of Hong Kong, Hong Kong, China

**Keywords:** cetuximab, chemotherapy, colorectal cancer, metastasis, meta-analysis

## Abstract

This meta-analysis used the database including PubMed, Medline, Cochrane Library, CNKI, Chinese-Cqvip, and Wanfang for randomized controlled trials (RCTs) to investigate the clinical effectiveness for combining cetuximab treatment with chemotherapy for treating metastatic colorectal cancer (mCRC). A total of 12 RCTs involved 7,108 patients with mCRC were included. The patients received chemotherapy with (3,521 cases) or without cetuximab (3,587 cases). Outcomes were overall survival (OS), progression-free survival (PFS), disease control rate (DCR), overall response rate (ORR), odd ratio (OR), and risk ratio (HR). Our results showed that the chemotherapy alone group has shorter OS, PFS, and ORR than the chemotherapy plus cetuximab group, with significant differences (PFS:HR = 0.77, 95% CI = 0.72–0.82, *P* < 0.00001; OS:HR = 0.88, 95% CI = 0.79–0.99, *P* = 0.03; ORR:OR = 1.79, 95% CI = 1.30–2.47; *P* = 0.0003). Results of subgroup analysis showed that cetuximab treatment prolonged PFS and OS in KRAS wild-type patients, with statistically significant differences (PFS:HR = 0.79, 95% CI = 0.65–0.95, *P* = 0.01; OS:HR = 0.85, 95% CI = 0.74–0.98, *P* = 0.02). Combining cetuximab with chemotherapy, the PFS and OS of wild-type KRAS patients and the ORR of all patients were significantly improved.

## Introduction

Globally, colorectal cancer (CRC) refers to one of top 3 fatal cancers, characterized with poor prognosis and high metastasis (1). However, the early symptoms of CRC are inconspicuous, and then around 15–25% of patients with CRC were diagnosed as advanced stage in initial check-up (2). As a result, 50% of patients with metastatic colorectal cancer (mCRC) are inoperable, thus having recurrence and metastasis after treatment (3). The traditional concept of mCRC chemotherapy is continuous treatment until the disease progresses, but the cumulative toxicity of the drug limits the continued use of chemotherapy. Therefore, measures to reduce toxicity during chemotherapy, prolong PFS and OS have become the key to treatment options. Thanks to the new generation of drugs with less toxicity and better targeting, they have gradually entered the clinical stage, and advanced tumor maintenance treatment has gradually demonstrated its clinical application advantages and have been successfully used in solid tumors, such as blood tumors, lung cancer and breast cancer (4–7). In the past two decades, with the progress of tumor molecular biology and new drug research, molecularly targeted drugs that specifically interfere with the biological behavior of tumors have gradually demonstrated clinical application advantages in tumor therapy due to their high selectivity and high therapeutic index. The NCCN guidelines recommend combining targeted drugs with mFOLFOX, FOLFIRI and XELOX as first-line treatments for mCRC (8). At present, there are mainly two types of drugs targeting EGFR, monoclonal antibodies and small molecule tyrosine kinase inhibitors (TKIs) have reached the mature stage of clinical application (9). Monoclonal antibodies are highly homogeneous antibodies derived from a single B-cell clone that targets only a specific epitope. Compared with chemotherapeutic drugs, monoclonal antibodies have the advantages of strong therapeutic effect, fewer adverse reactions, and high patient tolerance (10). Sirotnak et al. (11) have found that combining gefitinib with paclitaxel or docetaxel can significantly inhibit the growth of A431, LX-1, SK-LC-16, TSU-PR1, and PC-3 tumor cells as compared to single drugs; doxorubicin combined with gefitinib has 10-fold induction of A549 inhibitory effect. Ciardiello et al. (12) found that the combined effect of gefitinib and chemotherapeutic agents can significantly enhance apoptosis and synergistically inhibit tumor growth in mice with colon cancer (GEO) transplanted tumors and the tumor suppressive effect of gefitinib on tumor-bearing mice was reversible; but after the end of treatment, the tumor growth rate in the control group was still able to recover, while the tumors in the combination group started to grow slowly 4–8 weeks after the treatment.

Cetuximab, as a novel monoclonal antibody, was marketed in 2003 to target epidermal growth factor (EGFR) receptors and block intracellular signal transduction, thereby inhibiting proliferation of cancer cell (13). The main indication described in its drug insert (Merck, Germany) is mCRC which is resistant to chemotherapy with irinotecan-based drugs. Previous studies have found that mCRC is often accompanied with genetic mutations, such as K-Ras mutations, and the mutated mCRC is no longer regulated by EGFR (14, 15). At this point, the drugs that target EGFR will become ineffective. Therefore, EGFR inhibitors are only suitable for the treatment of K-Ras wild type mCRC. Cetuximab combined with chemotherapy is currently the standard protocol for first-line treatment of RAS wild-type mCRC patients (16). Several studies have shown that cetuximab significantly improves objective response rate (ORR), progression-free survival (PFS) and overall survival (OS) in patients with wild-type RAS mCRC, especially those with primary lesions on the left (17, 18). However, even in patients with left half colorectal cancer where the efficacy of cetuximab is dominant, about 30% of patients have failed (19). Besides, its clinical application time is relatively short. In addition, the clinical trial results of cetuximab in the treatment of mCRC in recent years are inconsistent. At present, there is no definitive agreement on its efficacy and adverse reactions. To further explore the use of cetuximab in mCRC, this article used meta-analysis combined with current published data to study the efficacy of cetuximab in combination with chemotherapy for mCRC treatment, so as to provide more reliable evidence-based medical evidence for its clinical use.

## Methods and Materials

### Search Strategy

We conducted database search and data analysis based on the criteria published in the Systematic Review and the Meta-analytical of Preferred Reporting Items (PRISMA) guidelines. Search for PubMed, Medline, Cochrane Library, CNKI, Chinese-Cqvip and Wanfang Database, time range from January 2004 to July 2018, extracting overall survival (OS), progression-free survival (PFS), disease control rate (DCR), overall response rate (ORR), odd ratio (OR), and risk ratio (HR) from literature reports related to mCRC patients result. The disease in these mCRC patients originated from KRAS wild-type or mutant colorectal cancer, and they were treated with or without cetuximab in a randomized controlled trial (RCT). The key words for searched were “metastatic colorectal cancer” (or “carcinoma” or “malignant tumor”) and cetuximab (or “erbitux”). In addition, there are some full text of the literatures were retrieved by reference of the retrieved literature. And all the articles had no limit for language. In addition, we conducted extensive searches and the articles were further verified in the list of references.

### Inclusion and Exclusion Criteria

#### Patient

Eligible patients were confirmed as mCRC and age 18 years or older with Eastern Cooperative Oncology Group performance status of 0, 1, or 2, and histologically proven stage III (any T, N1 or N2, M0) adenocarcinoma of the colon. Unlimited metastases, and no limit on the number of metastases; no geographical or gender restrictions; and renal and bone marrow hematopoiesis are normal. Life expectancy ≥12 weeks. All participants provided written informed consent before study enrollment and were required to submit blood and tumor tissue before randomization.

#### Intervention

Experimental group: cetuximab combined with chemotherapy; control group: chemotherapy.

#### Type of Design of Experime

The experiment should be a randomized controlled clinical trial. For studies with multiple intervention groups, relevant data are selected for inclusion. Exclude crossover trials and semi-randomized controlled trials by date or admission. When duplicate or repeating data appears in multiple reports, the data including the most comprehensive information is selected.

#### Outcomes

Overall survival (OS), progression-free survival (PFS), disease control rate (DCR), overall response rate (ORR), odd ratio (OR), and risk ratio (HR).

### Quality Assessment

**Figure 1:**
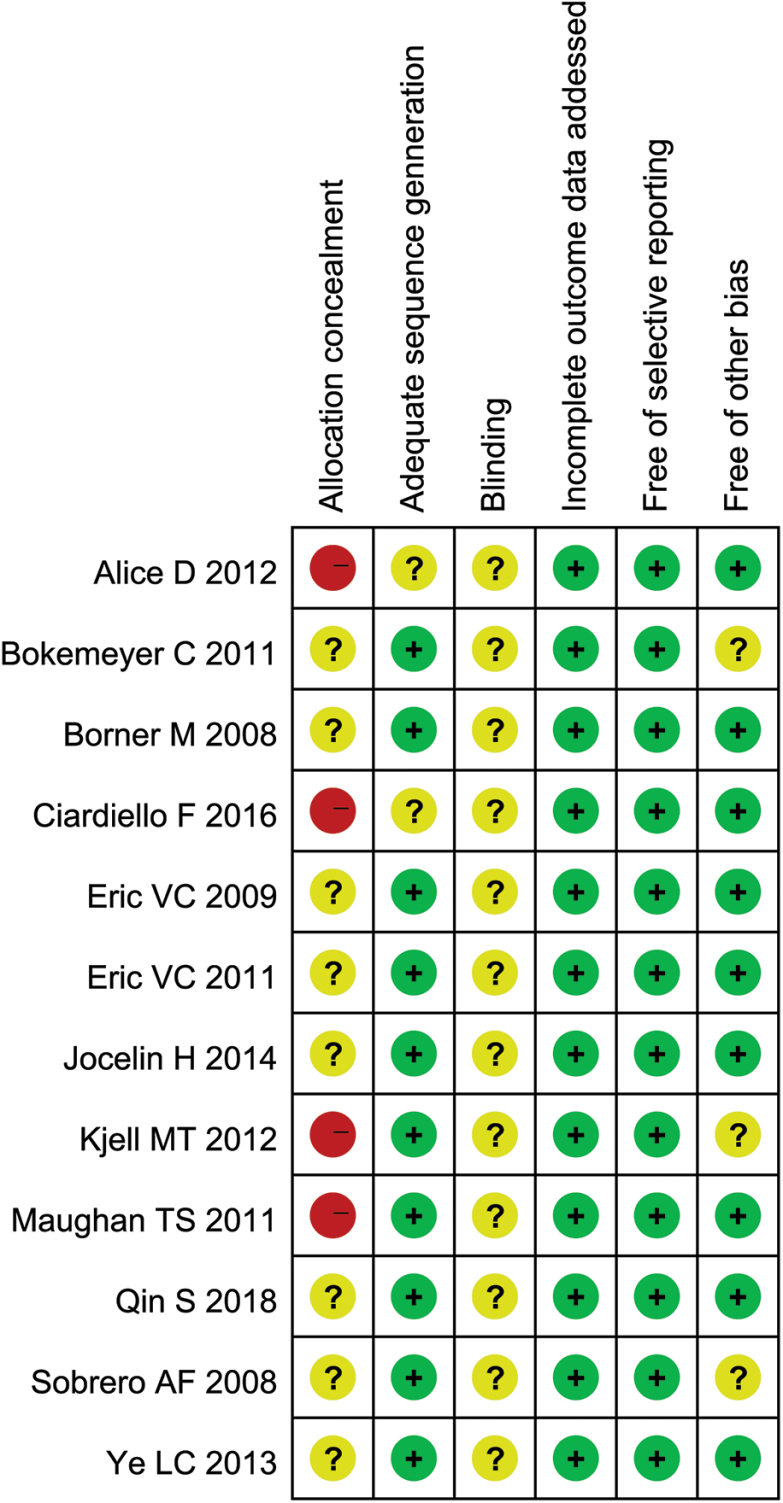
Bias assessment of 12 RCTs included.

The abstracts of all documents identified in the original search were screened and two researchers (Xiaoliu Liang and Yujia Liang) excluded studies that violated the inclusion criteria. Another author (Shiyuan Xie) post-evaluated the full-text article. If different opinions were generated, the third researcher was asked to evaluate such research and reach a consensus through discussion. Finally, the risk of selective biased project is recommended according to the Cochrane Handbook for Systematic Reviews of Interventions. Risks of biased items included: blinding, allocation concealment, proper sequence generation, incomplete outcome data, non-selective reporting, and other biases (Figure 1). If the article did not display the original data, contact the corresponding author of the study by email using a separately customized application form.

### Data Extraction

The following information was independently extracted from each study by two investigators (Xiaoliu Liang and Yujia Liang). When a disagreement occurs, it is resolved by consensus. Gather the following information from each eligible study: the first author's name, country, male/female ratio, age range, sample size, treatment regimens, ending index, publication year, intentionality therapy (Intention-to-treat, ITT) in patients with OS and PFS, HRs with corresponding 95% Cis, period of treatment, KRAS wild-type and KRAS-type mutations in OS and PFS patients, etc.

### Statistical Analysis

RevMan 5.0 software was used for all statistical analyses. Efficacy in a regimen of chemotherapy with mCRC in combination with cetuximab was evaluated based on data from RCT. DCR and ORR were analyzed by relative risk (RR) or odds ratio (OR); if RR > 1 or > 1, the experimental group (chemotherapy with cetuximab) was higher than the control group (chemotherapy without cetuximab); vice versa, if RR < 1 or < 1, the experimental group was lower than the control group. In addition, PFS and OS were the primary endpoints of pooled analysis, and expression of HR at the primary endpoint of each study was 95% CI. If the literature did not provide HR, it could be extracted according to the survival curve method (KM). Chi-square test and *I*^2^ statistics were used when estimating statistical heterogeneity. When *p* < 0.05 or *I*^2^ > 50%, a random effects model was used; otherwise a fixed effect model was used. If the heterogeneity was large, a descriptive analysis was performed. The stability of the test results was determined by sensitivity analysis if necessary.

## Results

### Selection and Characteristics of Study

**Figure 2:**
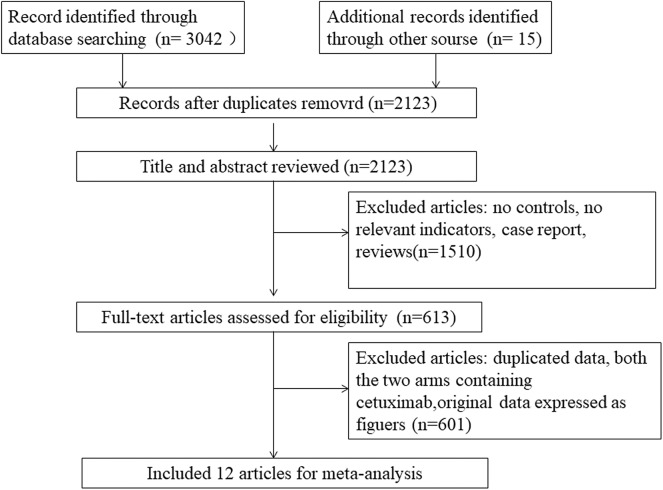
The flowchart of this study setting.

The overall flowchart of the study was shown in Figure 2. At the beginning, we included 3,057 potential studies. Due to the duplication, 934 publications were excluded. In addition, 1,510 articles with no controls, no relevant indicators, case reports, or no association with mCRC were excluded. Then, 601 articles with no relevant raw data, original data expressed as figures and duplicated data were excluded. Besides, study with the two arms containing cetuximab or the study with dual targeted drugs were also excluded. Finally, 12 articles met the requirements for this meta-analysis, involving 12 RCTs (20–31) (the experimental group: 3,587 cases and the control group: 3,521 cases). The characteristics of the 12 articles were shown in Tables 1,2.

**Table 1:** Characteristics of the RCT studies included in our meta-analysis.

			**Treatments**		**Age (median, range)**			
**References**	**Sex (male/female) Country**		**Control**	**Experiment**	**Control**	**Experiment**	**A period of treatment(d)**	**Duration**
Bokemeyer et al. (16)	181/156	Europe	FOLFOX	FOLFOX +cetuximab	60 (30-82)	62 (24-82)	14	Depending on disease progression and severity of adverse reactions
Huang et al. (28)	78/68	Europe	FOLFIRI	FOLFIRI +cetuximab	57 (25-82)	59 (30-82)	14	6 months
Dewdney et al. (29)	101/63	Multicenter	CAPOX	CAPOX +cetuximab	65 (28-79)	61 (28-79)	14	2 months
Van Cutsem et al. (27)	725/473	Europe	FOLFIRI	FOLFIRI +cetuximab	61 (19-84)	61 (22-82)	14	Depending on disease progression and severity of adverse reactions
Van Cutsem et al. (24)	725/473	Europe	FOLFIRI	FOLFIRI +cetuximab	61 (19-84)	61 (22-82)	14	Depending on the disease progression, the degree of adverse reactions or the informed consent was withdrawn
Tveit et al. (31)	220/159	Multicenter	FLOX	FLOX +cetuximab	61.2 (29.9–74.8)	60.8 (24.1–74.4)	14	Depending on disease progression and severity of adverse reactions
Ye et al. (26)	88/50	Europe or North America	FOLFOX or FOLFIRI	FOLFOX or FOLFIRI +cetuximab	59 (35–75)	57 (26–75)	14	Depending on the reaction after liver metastasis of cancer, disease progression or the degree of adverse reactions
Borner et al. (20)	44/30	Multicenter	CAPOX	CAPOX+cetuximab	63 (47–80)	60 (37–81)	21	4.5 months or disease progression
Maughan et al. (21)	1068/562	UK	CAPOX or FOLFOX	CAPOX or FOLFOX +cetuximab	63 (56–69)	63 (58–70)	14	Depending on disease progression
Ciardiello et al. (23)	72/81	Chinese	FOLFOX	FOLFOX+cetuximab	49–59	49–59	14	Depending on the disease progression, the degree of adverse reactions or the informed consent was withdrawn
Qin et al. (22)	266/127	Multicenter	FOLFOX	FOLFOX +cetuximab	56 (21-78)	56 (21–83)	14	Depending on disease progression and severity of adverse reactions
Sobrero et al. (25)	816/482	Multicenter	Irinotecan	Irinotecan +cetuximab	62 (21-90)	61 (23–85)	21	Depending on disease progression and severity of adverse reactions

**Table 2:** Characteristics of the RCT studies included in our meta-analysis.

**References**	**Number of cases**		**HR (95%CI)**	
	**Control**	**Experiment**	**PFS**	**OS**
Bokemeyer et al. (16)	168	169	0.931(0.705,1.23)	1.105(0.791,1.303)
Huang (28)	106	40	0.53(0.26,1.1)	0.45(0.17,1.16)
Dewdney (29)	81	83	0.81(0.45,1.44)	0.53(0.26,1.10)
Van Cutsem (27)	599	599	0.851(0.726,0.998)	0.878(0.774,0.995)
Van Cutsem (24)	599	599	0.85(0.72,0.99)	0.93(0.81,1.07)
Tveit (31)	185	194	0.89(0.72,1.11)	1.06(0.83,1.35)
Ye et al. (26)	68	70	0.6(0.41, 0.87)	0.54 (0.33, 0.89)
Borner et al. (20)	37	37	NR	NR
Maughan et al. (21)	815	815	NR	NR
Ciardiello et al. (23)	79	74	0.56(0.33, 0.94)	0.57(0.32, 1.02)
Qin et al. (22)	200	193	0.69(0.54, 0.89)	0.76(0.61, 0.96)
Sobrero et al. (25)	650	648	0.692(0.617, 0.776)	0.975(0.854, 1.114)

### Meta-Analysis of DCR and ORR

**Figure 3:**
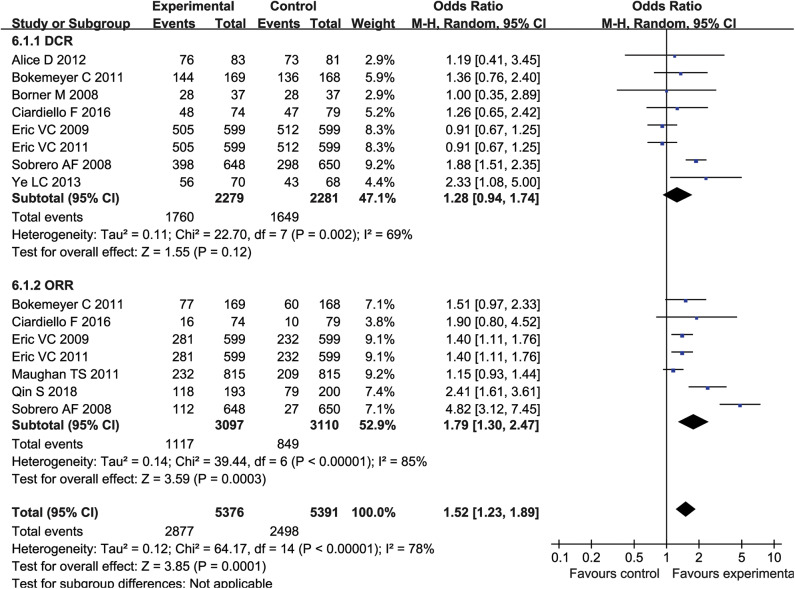
The ORR and DCR of forest plots with fixed effect model.

Eight studies (4,560 patients) reported DCR, and seven studies (6,207 patients) reported ORR. The results showed heterogeneity between studies (DCR: *P* = 0.002, *I*^2^ = 69%; ORR: *P* < 0.00001, I2 = 85%) (Figure 3), so a random effects model was used for meta-analysis. Meta-analysis showed no significant difference in DCR between the experimental group and the control group (OR = 1.28, 95% CI = 0.94-1.74, *P* = 0.12) (Figure 3). However, patients receiving combination therapy with cetuximab had higher ORR (OR = 1.79, 95% CI = 1.30–2.47; *P* = 0.0003) (Figure 3).

### Meta-Analysis of PFS

**Figure 4:**
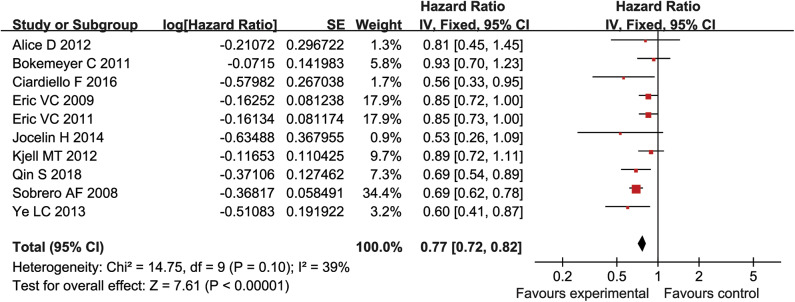
The PFS of forest plots with fixed effect model.

PFS was reported in ten studies (5,404 patients) and there was no statistical heterogeneity between each study (*P* = 0.1, *I*^2^ = 39%) (Figure 4). The log HR values of PFS were analyzed by fixed effect model and inverse variance method. The results suggested that the PFS of experimental group was significantly longer than that of control group (HR = 0.77, 95% CI = 0.72–0.82, *P* < 0.00001) (Figure 4).

### Meta-Analysis of OS

**Figure 5:**
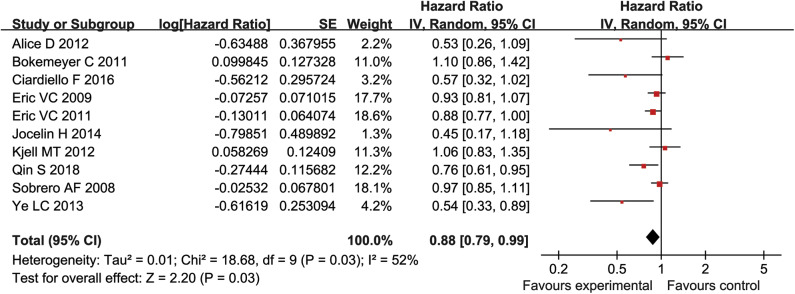
The OS of forest plots with fixed effect model.

There were 10 studies reported OS (5,404 patients). There was heterogeneity between the studies (*P* = 0.03, *I*^2^ = 52%) (Figure 5). Therefore, for the log HR values of the OS, a fixed effect model and an inverse variance method were used for meta-analysis. Analysis showed that the experimental group had significant advantages in improving OS, as compared to the control group (HR = 0.88, 95% CI = 0.79–0.99, *P* = 0.03), (Figure 5).

### Subgroup Analysis

**Figure 6:**
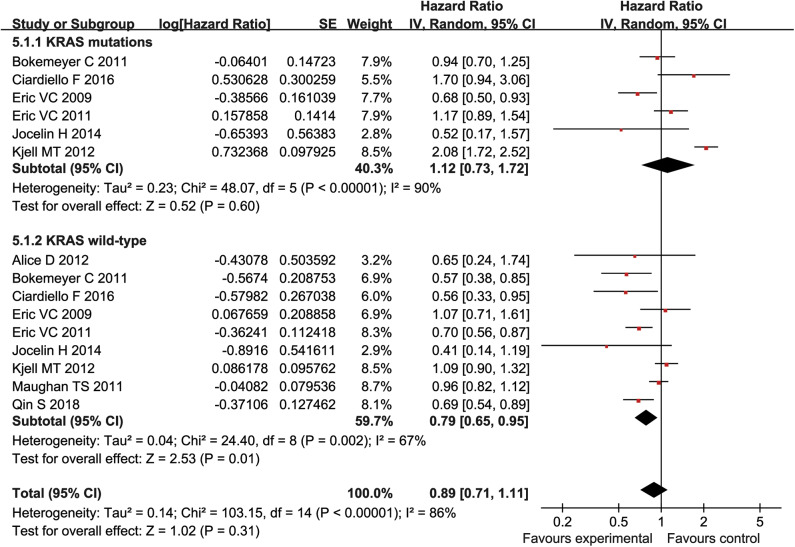
OS forest plot.

**Figure 7:**
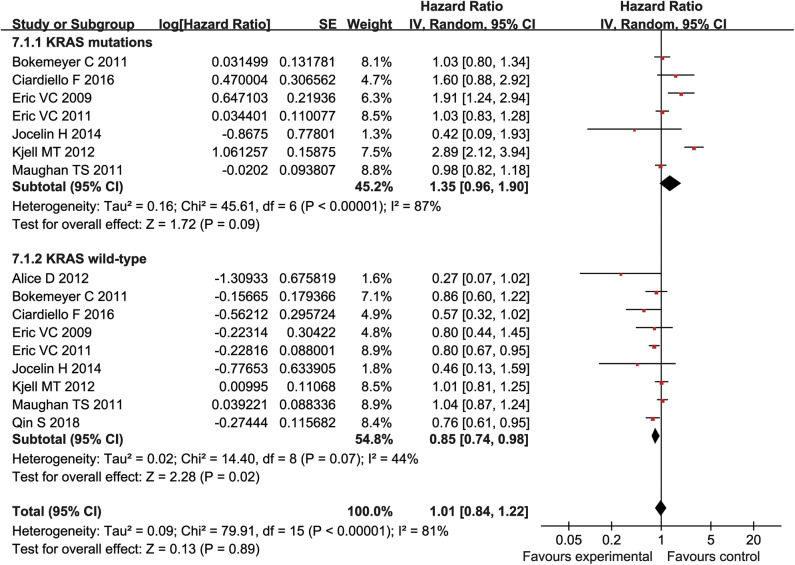
PFS forest plot.

Patients were divided into mutant KRAS and wild type KRAS according to their KRAS genotypes. The HR with 95% CI were extracted from KRAS wild-type and mutant KRAS of patients in each study, followed by the subgroup analysis. Our result showed that cetuximab can significantly prolonged PFS and OS in patients with KRAS wild type (PFS:HR = 0.79, 95% CI = 0.65–0.95, *P* = 0.01; OS:HR = 0.85, 95% CI = 0.74–0.98, *P* = 0.02) (Figures 6, 7), but there was no significant change of PFS and OS in patients with KRAS mutations when chemotherapy was used in combination with cetuximab (PFS:HR = 1.12, 95% CI = 0.73–1.72), *P* = 0.6; OS:HR = 1.35, 95% CI = 0.96–1.90, *P* = 0.09) (Figures 6, 7).

### Publication Bias

**Figure 8:**
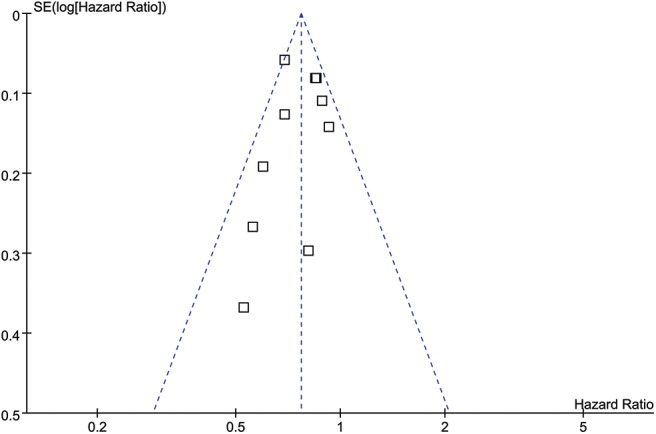
Funnel plot.

The PFS was used as the index to draw the inverted funnel plot. The result showed that the arrangement of each study around the Central Line was not completely symmetrical, suggesting that there was a certain publication bias in the included articles (Figure 8).

## Discussion

A total of 12 studies involving 5,404 patients were included in our meta-analysis. Our analysis used a large number of enrolled patients, strict inclusion and exclusion criteria, and similar outcome indicators among studies. Our results showed that cetuximab could significantly prolong PFS and OS in mCRC patients with wild type KRAS, but did not remarkably improve PFS and OS in patients with KRAS mutations. This result was concordant to Wang li's finding that reported the relationship between KRAS gene polymorphism and targeted therapy for colorectal cancer (32). They concluded that cetuximab treatment was ineffective if KRAS gene codon 12 and 13 were mutated. While a meta-analysis conducted by Zhou et al. found that oxaliplatin-based chemotherapy combined with cetuximab or other anti-EGFR monoclonal antibodies could not prolong the survival of mCRC patients (33). It could be explained by the use of different chemotherapeutic drugs. Because in our 12 RCTs studies, five of the studies used folfiri and irinotecan, instead of oxaliplatin-based chemotherapy. For the DCR of intention to treat (ITT) patients, the efficacy of chemotherapy drugs combined with cetuximab was comparable to that of chemotherapy drugs alone, which was consistent with the conclusion of the meta-analysis of 12 RCTS conducted by Wang et al. (34). Our result also indicated that the ORR of the experimental group was significantly higher than that of the control group, which was consistent with the meta-analysis of Ye et al. (26). Additionally, Qin et al. (22) and Angeles et al. (14) obtained a positive result through RCT, suggesting that the use of cetuximab can be benefit to mCRC patients, while RCT conducted by Yu et al. (19), Sirotnak et al. (11) came to a contrary conclusion. Therefore, there is no consensus on the effective therapeutic significance of cetuximab in mCRC patients with ITT. This may be caused by different sample sources and different experimental methods among different studies.

The KRAS gene polymorphism is a biomarker that reflects changes in EGFR receptors and is associated with the efficacy of cetuximab (27). Foreign CRC diagnostic and therapeutic guidelines suggested that the genetic status of KRAS should be tested before CRC patients treated with cetuximab, while cetuximab is indicated for CRC patients with wild-type KRAS (28). To further elucidate the efficacy relationship between cetuximab and KRAS genotyping, this study performed a meta-analysis of OS and PFS in mCRC patients with wild-type or mutant KRAS, and the conclusions were similar to the finding from Dewdney et al. (29). Another RCT study performed by Christos SK indicated that the use of cetuximab is more effective in patients with wild-type KRAS than that of patients with KRAS mutations (30). References included in this study were all from foreign databases. In addition, the title and abstract attributives of the literature searched in this study were in English, and the literature published in other languages were not included, so there was a database retrieval bias. In the process of literature screening, software screening and manual screening are adopted. The software screening is simple and easy to operate, but it is mechanical, with low recognition ability and the possibility of omission. Manual screening has a large workload and high recognition ability, but it is possible to wrongly reject some negative conclusions, both of which will lead to bias in literature screening. Inverted funnel plot analysis showed that the included studies were not completely symmetrical, which also suggested some publication bias. In addition, due to the designs of the study, there is no information on the number of prior treatments patients have completed, so the information on successful treatment outcome is still not firmly confirmed.

## Conclusions

In summary, compared with chemotherapy alone, combined with cetuximab can significantly prolong PFS and OS in mCRC patients. Limited by the quality and sample size of included studies, this conclusion needs to be verified by a larger sample of RCTs with strict design and long-term follow-up.

## Data Availability Statement

The datasets generated for this study are available on request to the corresponding author.

## Author Contributions

RL and ML collected and assayed the data. RL, XL, and LY conducted statistical analysis and plotted figures. XL and MS discussed the data. KL designed this study, discussed the findings and drafted the manuscript.

## Conflict of Interest

The authors declare that the research was conducted in the absence of any commercial or financial relationships that could be construed as a potential conflict of interest.
